# Acalabrutinib and Obinutuzumab for Chronic Lymphocytic Leukemia Complicated With Peripheral Neuropathy

**DOI:** 10.7759/cureus.74703

**Published:** 2024-11-28

**Authors:** Taro Edahiro, Hiroshi Ureshino, Masahiro Nakamori, Takero Shindo, Tatsuo Ichinohe

**Affiliations:** 1 Hematology and Oncology, Research Institute for Radiation Biology and Medicine, Hiroshima University, Hiroshima, JPN; 2 Clinical Neuroscience and Therapeutics, Hiroshima University, Hiroshima, JPN

**Keywords:** acalabrutinib, chronic lymphocytic leukemia, obinutuzumab, peripheral neuropathy, vasculitis

## Abstract

A 40-year-old man presented to our hospital with subacute progressive muscle weakness in the limbs and leukocytosis. Subsequently, the patient was diagnosed with chronic lymphocytic leukemia (CLL) complicated by peripheral motor neuron neuropathy (axonopathy). Serology test for anti-ganglioside GM2 IgG antibody was positive, whereas paraneoplastic syndrome-related and anti-myelin-associated glycoprotein antibodies were not detected. There was no evidence of advanced-stage CLL. Intravenous immunoglobulin and steroid pulse therapy were initiated with slight symptom amelioration. Acalabrutinib and obinutuzumab were administered, and his symptoms gradually improved. Hence, acalabrutinib and obinutuzumab may have been active in patients with CLL complicated by peripheral neuropathy.

## Introduction

Chronic lymphocytic leukemia (CLL) is a mature B-cell neoplasm [1], and its clinical presentation is generally indolent (more than 80% of cases are diagnosed at an early stage); thus, patients with early-stage CLL generally do not need anti-leukemic treatment [2,3]. If CLL progresses, patients may experience symptoms such as fatigue, fever, lymphadenopathy (swollen lymph nodes), weight loss, and other related issues.

The B-cell receptor (BCR) signaling pathway plays an important pathogenic role in CLL [4]. Additionally, Bruton’s tyrosine kinase (BTK) inhibitors disrupt BCR signaling in CLL cells, exerting anti-leukemic effects. Ibrutinib, a first-generation BTK inhibitor, is the standard of care for symptomatic CLL [5]. Acalabrutinib, a second-generation BTK inhibitor, along with obinutuzumab, is also effective in these patients [6].

CLL is often complicated by autoimmune diseases [7] such as autoimmune hemolytic anemia (approximately 5-10% of CLL patients) and immune thrombocytopenia (approximately 2-5% of CLL patients), associated with multifactorial aberrant humoral, cellular, and innate immunity. However, neurological complications are not common during the course of CLL [8] and their pathogenesis has not been fully elucidated.

Herein, we report the case of a patient with CLL complicated by peripheral motor neuron neuropathy, who was successfully treated with acalabrutinib and obinutuzumab.

## Case presentation

A 40-year-old man visited our hospital with a one-year history of subacute progressive limb muscle weakness in June 2022. He first noticed muscle weakness in his right limbs in the autumn of 2021. He monitored the symptoms before visiting the hospital, during which time the weakness progressed to involve both his upper and lower limbs, particularly affecting his fingers and toes. By the time he visited our hospital, his symptoms had worsened to the extent that he was unable to open a bottle cap and could not walk without using a handrail. Manual muscle testing revealed significant weakness, especially in the fingers and toes, with the following results: abductor pollicis brevis 2/2, abductor digiti minimi 1/1, and extensor digitorum brevis 2/2. Despite this, deep tendon reflexes of the biceps, triceps, brachioradialis, patellar, and Achilles tendons were normal. No signs of optic neuropathy were observed.

**Figure 1:**
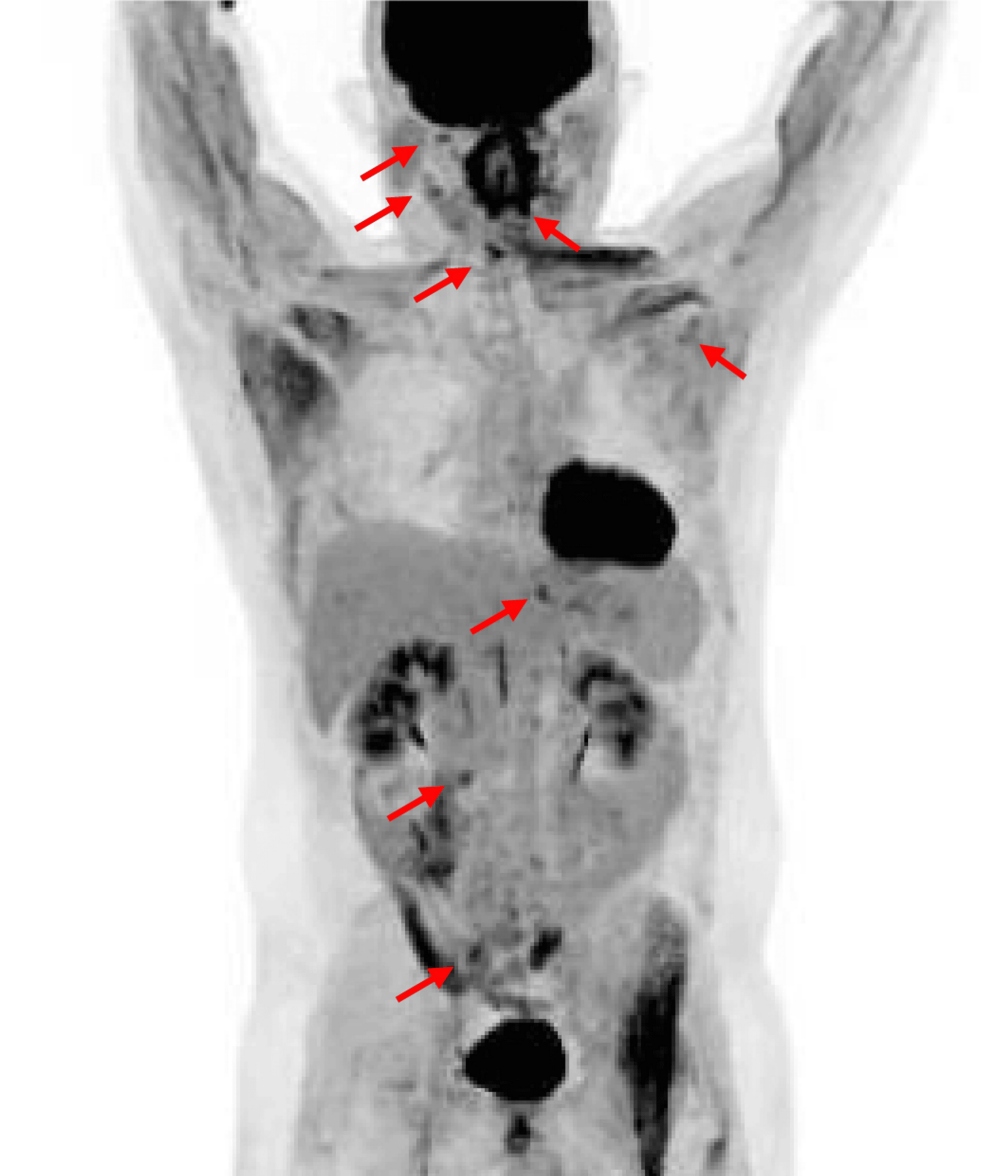
FDG positron emission tomography/computed tomography Mildly increased FDG uptake in the systemic lymph nodes was observed (red arrows). FDG, fluorodeoxyglucose.

A complete blood count revealed elevated white blood cells (13,840/μL) with abnormal lymphocyte count (43%), and normal hemoglobin and platelet levels. Flow cytometric analysis revealed that the abnormal lymphocytes were positive for CD5, CD19, CD20, and CD23. A bone marrow aspirate revealed an increased percentage of mature lymphocytes (70.8%) with no detectable obvious morphological abnormalities. G-banded metaphase analysis showed a normal karyotype (46, XY [20/20]), while immunoglobulin heavy-chain (IgH) gene rearrangement was detected, and fluorescence in situ hybridization showed no TP53 and ATM deletions. The mutational status of the immunoglobulin heavy-chain variable region (IGHV) gene has not been tested in general clinical practice in Japan. [18F]-Fluorodeoxyglucose (FDG) positron emission tomography/computed tomography showed mildly increased FDG uptake in the systemic lymph nodes (Figure 1). A diagnosis of CLL (revised Rai stage 0) was made.

**Figure 2:**
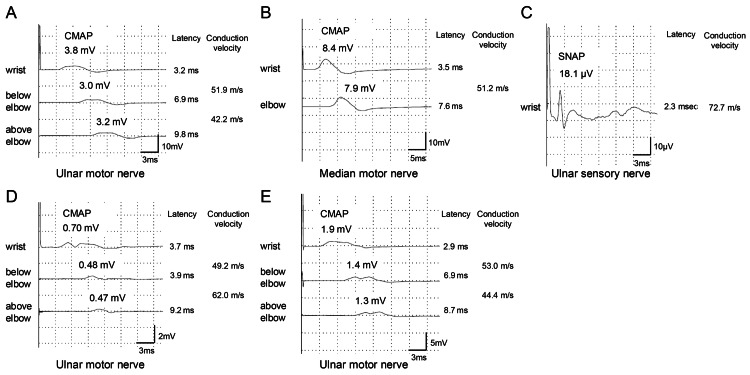
The results of nerve conduction studies. A. The motor conduction study of the right ulnar nerve (at the first diagnosis). B. The motor conduction study of the right median nerve (at the first diagnosis). C. The sensory conduction study of the right ulnar nerve (at the first diagnosis). D. The motor conduction study of the right ulnar nerve at the end of the methylprednisolone therapy (during maximum symptom exacerbation). E. The motor conduction study of the right ulnar nerve at the end of acalabrutinib and obinutuzumab combination therapy. CMAP, compound muscle action potential; SNAP, sensory nerve action potential.

A neurological examination revealed asymmetrical distal weakness (motor neuron deficit) in the upper and lower limbs. Nerve conduction studies (NCS) revealed that the latency period and conduction velocity were normal, whereas the compound muscle action potential (CMAP) of the right ulnar nerve was low (Figure 2A). The sensory nerves in the latency period, conduction velocity, and amplitude were normal (Figure 2B). The damaged nerves presented a patchy and asymmetrical pattern in the limbs (especially the right ulnar nerve). Ultrasound examination of the peripheral nerves revealed skipped thickness of the bilateral median nerves and left ulnar nerve. Based on the NCS results, axonopathy was diagnosed. Considering the presence of multiple mononeuropathy induced by axonal damage, neuropathy-associated vasculitis could have occurred in this patient. 

Serum anti-ganglioside IgG GM2 antibody was positive, while serum perinuclear and cytoplasmic anti-neutrophil cytoplasmic antibody (ANCA), cryoglobulin, paraneoplastic syndrome-related antibodies (AMPH, CV2, PNMA2, Ri, Yo, Hu, recoverin, SOX1, titin, zic4, GAD65, and Tr), and anti-myelin-associated glycoprotein antibody were negative. 

**Figure 3:**
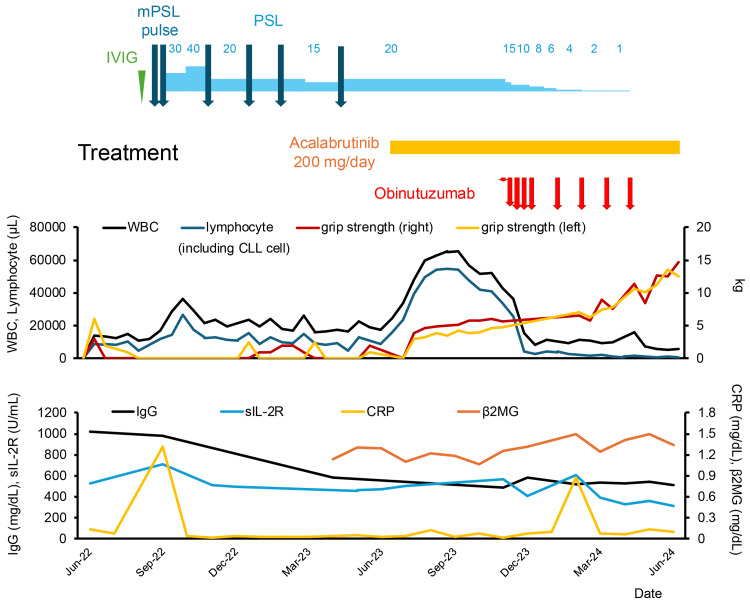
Clinical course. IVIG, intravenous immunoglobulin; mPSL, methylprednisolone; PSL, prednisolone; WBC, white blood cell; IgG, immunoglobulin G; sIL-2R, soluble interleukin 2 receptor; CRP, C-reactive protein; β2MG, beta-2 microglobulin.

Serum soluble interleukin-2 receptor (525 U/mL), β2 microglobulin (1.14 mg/L), C-reactive protein (0.13 mg/dL), and IgG (1021 mg/dL) levels were not elevated (Figure 3). CLL (revised Rai stage 0) complicated by peripheral neuropathy (PN) (axonopathy) was diagnosed. Intravenous immunoglobulin (IVIg) was initiated; however, the neuropathy did not ameliorate. Subsequently, steroid pulse therapy (methylprednisolone 1000 mg for three days) was initiated, and his neurological symptoms slightly ameliorated. However, the symptoms relapsed when the corticosteroid dose was tapered. The results of CMAP revealed that the amplitude was very low even after steroid pulse therapy (Figure 2D). The nerve biopsy was not performed.

The neurological symptoms could be attributed to vasculitis induced by CLL invasion depending on an asymmetrical axonopathy; therefore, acalabrutinib (200 mg daily) and obinutuzumab (1000 mg) were initiated. Lymphocyte counts in the peripheral blood rapidly decreased after the first dose of obinutuzumab, and this treatment combined with acalabrutinib therapy resulted in gradual clinical amelioration (e.g., grip strength increased from approximately 0 kg to over 10 kg in both hands; Figure 3). The patient achieved complete remission four months after treatment initiation. The serum IgG level gradually decreased following the initiation of corticosteroids, and subsequently after starting acalabrutinib and obinutuzumab (Figure 3). The results of CMAP revealed that the right ulnar nerve also increased (Figure 2D, 2E). The patient returned to work and visited our hospital regularly, while acalabrutinib is still ongoing.

## Discussion

Here, we report a case of CLL complicated by PN.

PN is a rare complication of CLL [8]; only 19 of 816 (2.3%) patients with CLL develop PN. The mechanisms of CLL-associated PN include direct leukemic infiltration of the peripheral nerve system [9] or autoimmune mechanisms such as chronic inflammatory demyelinating polyradiculoneuropathy (CIDP), Guillain-Barré syndrome (GBS) and its variants, Miller-Fisher syndrome, and other inflammatory neuromuscular disorders [9,10]. CIDP is relatively common in patients with CLL [8]. In contrast, subacute progressive motor neuropathy (axonopathy type) is mainly caused by vasculitis [11] which could be either antibody- (e.g., ANCA), immune complex-, cell-, or ischemia-mediated vasculitis (microvasculitis) [12].

In the present case, subacute axonopathy complicated by CLL progressed, and no autoantibodies (except for anti-GM2 antibodies that cause demyelinating disorders) were detected, indicating that PN might be a CLL cell-mediated vasculitic neuropathy. 

The use of corticosteroids is recommended for the treatment of vasculitic neuropathy; however, our patient responded only partially to this treatment. Nevertheless, obinutuzumab and acalabrutinib combination therapy, which directly depleted CLL cells, was strongly active not only against CLL cells but also effectively treated vasculitic neuropathy, suggesting that the latter was possibly associated with CLL cell-mediated vasculitis. Although direct CLL cell invasion of the optic nerve sheath in patients with CLL and CNS involvement has been reported [13], pathological confirmation of direct CLL invasion was not achieved in the present case.

Gangliosides (GM1, GM2, GM3, GD2, and GD3) are cell membrane components that are concentrated in the peripheral nervous system and are the major target antigens associated with immune-mediated demyelinating neuropathies [9,10]. In the present case, anti-GM2 IgG was detected, while the type of neuropathy did not indicate demyelinating diseases such as GBS and CIDP. Moreover, the clinical course and the absence of response to IVIg suggested that the neuropathy might not have been caused by anti-GM2 autoantibodies.

The combination of acalabrutinib and obinutuzumab is highly active and well-tolerated in patients with CLL. This combination may result in a high rate of molecular remission compared with acalabrutinib monotherapy [6]. Furthermore, anti-CD20 monoclonal antibody therapy may be active for CLL patients with PN in terms of the improvement of neurological symptoms [14]. In the present case, the patient achieved complete remission without severe adverse events four months after the initiation of treatment. The addition of obinutuzumab demonstrated early hematological improvement and accelerated the improvement of neurological symptoms. These results suggest that the combination of acalabrutinib and obinutuzumab may be a reasonable treatment for CLL complicated by neuropathy.

## Conclusions

To the best of our knowledge, this is the first case report describing a patient with CLL complicated by PN who was successfully treated with acalabrutinib and obinutuzumab. The combination of acalabrutinib and obinutuzumab may be effective for CLL complicated by neuropathy even in early-stage CLL.
